# Determination of Retention Behavior and p*K*_a_ Values of Some Phenothiazines with Green Chemistry Approach

**DOI:** 10.1021/acsomega.5c05625

**Published:** 2025-10-22

**Authors:** Zehra Öztürk, Ebru Çubuk Demi̇ralay, Hülya Yilmaz

**Affiliations:** † Department of Chemistry, Faculty of Engineering and Natural Sciences, 52994 Suleyman Demirel University, Isparta 32260, Turkey; ‡ Department of Basic Pharmaceutical Sciences, Faculty of Pharmacy, 52994 Suleyman Demirel University, Isparta 32260, Turkey; § Nanotechnology Research and Application Center (SUNUM), Sabancı University, Istanbul 34956, Turkey

## Abstract

In this study, environmentally friendly and conventional analytical methods based on green chemistry principles were developed to determine the dissociation constant (p*K*_a_) values of phenothiazine derivatives promethazine, promazine, and trimeprazine by reverse-phase liquid chromatography (RPLC). The retention values of the compounds were determined at a constant flow rate of 1 mL/min using mobile phases containing different concentrations of ethanol (EtOH) and acetonitrile (ACN) in the pH range of 5.5–11.5. The p*K*_a_ (_s_^s^p*K*_a_) values of the compounds in water–EtOH and water–ACN (50, 55, 60%, v/v) binary mixtures were determined at two different temperatures (25, 37 °C) using a Kinetex EVO C18 Core–Shell column (150 × 4.6 mm ID, 5 μm). Using the _s_^s^p*K*_a_ values of the compounds in the mobile phase environment and the macroscopic constants (mole fraction, dielectric constant) of the solvent used, the aqueous p*K*_a_ (_w_^w^p*K*_a_) values of these compounds, which are moderately soluble in water, were calculated. Accordingly, for promethazine, promazine, and trimeprazine, the values were calculated as 9.821, 10.407, and 10.110 at 25 °C and 9.787, 9,836, and 10.027 at 37 °C, respectively. The Green Solvent Selection Tool, the Analytical Greenness Metric Approach, the Green Analytical Procedure Index, and the Blue Applicability Grade Index were applied to assess the environmental impacts of both the green and conventional RPLC methods developed in the study.

## Graphical abstract

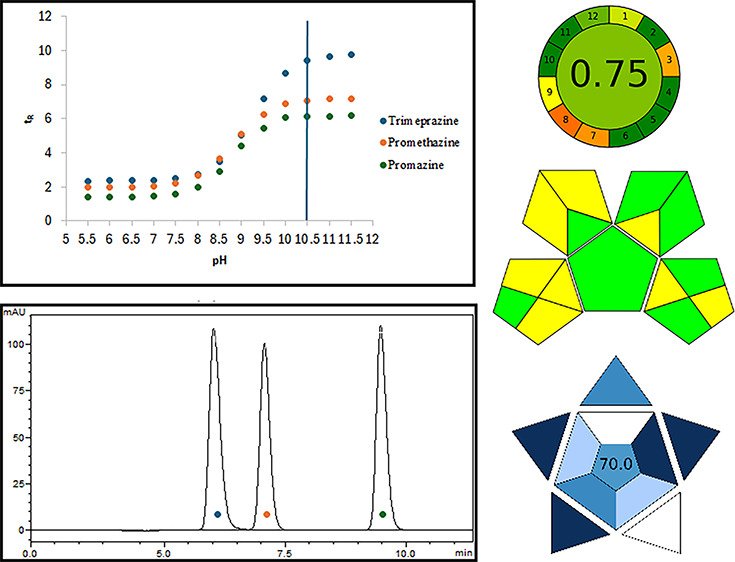

## 1 Introduction

Histamine H_1_ receptor (H1R) antagonists have long been widely used in treating allergic inflammations by competitively blocking histamine at H1R. These antagonists are classified into six groups based on their chemical structure, one of which is phenothiazines, known for their significant antiemetic, sedative, and antimuscarinic effects.^1^ Phenothiazines feature a tricyclic structure consisting of two benzene rings connected by a sulfur atom and a nitrogen atom, as shown in Figure 1d. Substitution of an electron-withdrawing group at position 2 (R2) enhances the antipsychotic activity of phenothiazines. The nature of the substituent at position 10 (R10) plays a role in pharmacological activity.^2^ Phenothiazines with antipsychotic, antihistamine, or anticholinergic effects have three carbon bridges between the 10-position of the central ring and the first amino nitrogen atom of the side chain at this position.^2^ Neuroleptic properties of phenothiazines may be affected by the nature of the chain in the 10-position, an amino group, or R2 substitution. For this reason, phenothiazine derivatives are used in various therapies due to their structure. Figure 1a–c show the chemical structures of promethazine, promazine, and trimeprazine, respectively.

**Figure 1:**
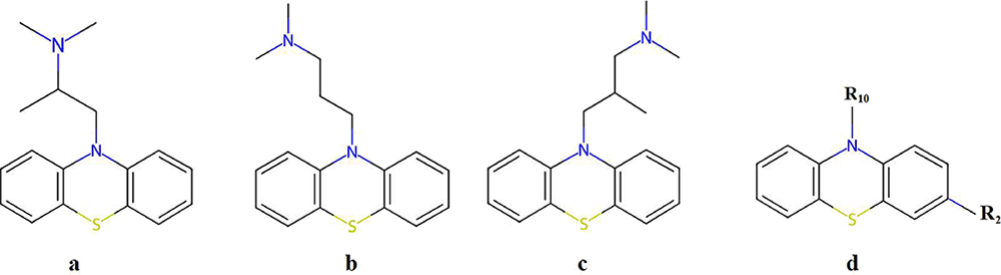
Chemical structures of (a) promethazine, (b) promazine, (c) trimeprazine, and (d) phenothiazine ring.

Understanding the action mechanism of active pharmaceutical ingredients (API) is crucial for linking their biological effects to their physical and chemical properties. Comprehensive knowledge of the chemical changes in the API from administration to excretion is essential, as the active ingredient’s physicochemical parameters determine these changes. As a key determinant of a drug’s pharmacokinetic properties, the dissociation constant (p*K*_a_) of its functional groups critically influences absorption and bioavailability, making this physicochemical parameter essential for designing both drug delivery vehicles and excipients.^3,4^ Various analytical methods, including UV/fluorescence spectrophotometry, potentiometry, capillary electrophoresis (CE), and reverse-phase liquid chromatography (RPLC), are widely used to determine the p*K*_a_ values of compounds. Among these, RPLC is particularly preferred due to its advantages, such as ease of application, reproducibility, accuracy, sensitivity, and specificity of the developed methods.^4,5^ Separation in RPLC is possible because of the different interactions of compounds with the stationary and mobile phases in the liquid chromatography system. Differences in the affinity of compounds for the stationary phase enable separation. Therefore, column selection in the RPLC method is crucial. Moreover, in RPLC, the retention behavior of analytes, influenced by their acid–base properties, depends on the organic solvent concentration in the mobile phase, the pH of the mobile phase, and the column temperature, which serve as key optimization parameters.^5,6^ Ionized and molecular forms of analytes have different retention properties within the RPLC column. By measuring the retention time values (*t*_R_/*k*) of these forms at varying mobile phase pH values, it is possible to analyze how retention changes correspond to changes in analyte ionization. Determining these retention changes with respect to pH allows the p*K*_a_ value of a compound to be determined. Therefore, using these approaches in RPLC, the p*K*_a_ values of ionized compounds can be reliably determined.^7–14^

For the determination of p*K*_a_ values, it is essential to first establish the solubility of the compounds. Water is typically the solvent of choice for many analysts due to its environmentally friendly nature. However, for compounds with low aqueous solubility, _s_^s^p*K*_a_ measurements are often performed in mixtures of water and an organic solvent. In RPLC analyses, organic solvents, such as acetonitrile (ACN), methanol (MeOH), and tetrahydrofuran (THF), are commonly used. These solvents are preferred because they are transparent in the relevant UV range, have strong abilities to dissolve hydrophobic compounds, and possess lower UV cutoff values.^15^

The widespread use of these conventional organic solvents in large amounts leads to significant waste production. As a result, more researchers agree that the use of such harmful solvents should be reduced or replaced with more environmentally friendly options. In response, the green RPLC approach has been developed and is gaining more interest in both routine analyses and research and development settings.^9,16^ Green RPLC methods favor the use of ethanol (EtOH) over the more commonly used conventional organic solvents.^16,17^ EtOH is considered one of the most environmentally friendly organic solvents, making it particularly well suited to sustainable liquid chromatography. Compared to ACN and MeOH, EtOH has a lower vapor pressure, resulting in less evaporation and consequently lower inhalation risks, which contribute to its lower toxicity. According to the Snyder classification, EtOH and MeOH belong to the same group in terms of solvent selectivity. However, the higher viscosity of EtOH compared to ACN and MeOH can increase backpressure in RPLC systems. As a result, the *t*_R_ values of analytes can be shorter when using EtOH compared to other solvents.^18^

In drug research and development, determining the aqueous p*K*_a_ (_w_^w^p*K*_a_) of compounds is crucial. However, even if a compound has sufficient water solubility, directly determining its p*K*_a_ using the RPLC method in a 100% aqueous environment is often not preferred due to potential issues, such as inconsistent retention times, peak asymmetry, and phase collapse in the RPLC column. To address this, one can first determine the _s_^s^p*K*_a_ values of compounds in hydroorganic mixtures since the amount of organic modifiers directly affects this value. The _w_^w^p*K*_a_ value can be estimated using the linearity between the macroscopic parameters of the organic solvent (e.g., X, ε) and the _s_^s^p*K*_a_ values.^3,19^ There is limited data in the literature regarding the _w_^w^p*K*_a_ values of promethazine, promazine, and trimeprazine.^20–24^

The primary objective of this research is to systematically analyze the p*K*_a_ values and optimal separation conditions of selected compounds using the green RPLC approach, a process that has not been previously determined. For this purpose, RPLC analyses of promethazine, promazine, and trimeprazine were conducted in binary mixtures of EtOH and water containing three different concentrations (v/v%) of EtOH in the mobile phase at various pH values. To demonstrate the retention behavior and p*K*_a_ changes due to solvent differences, analyses of the investigated compounds were performed in binary mixtures of ACN and water containing the toxic solvent ACN, which is used in conventional RPLC analyses. In the presented study, RPLC was used to gain a better understanding of how the ionization of selected compounds is affected by pH changes. While accurate pH measurement in water–organic solvent mixtures is crucial for all analyses, it presents both conceptual and practical challenges. This is due to the fundamental incomparability of the conventional pH values (pH_s_) between solvents. In these binary mixtures, pH measurements of acid–base properties are determined by the solvent’s autoprotolysis constant, and each solvent has its own solvent-specific pH scale, called pH_s_. In this study, pH standardization was considered crucial to accurately determine the mobile phase pH.^25^ This approach increases the reliability of the data obtained from the RPLC analysis of these selected phenothiazine derivative compounds. The data obtained in this study are the first in the literature. Additionally, to determine the suitability of solvents used in the developed method for green chemistry analysis, Green Solvent Selection Tool (GSST), Analytical Greenness Measurement (AGREE), Green Analytical Procedure Index (GAPI), and a specially designed tool to determine the environmental friendliness score of RPLC conditions, Blue Applicability Grade Index (BAGI) were used.^26–30^

## 2 Materials and Methods

### 2.1 Chemicals and Reagents

The chemicals used in the developed method were of analytical grade and were used as received without any further purification. Promethazine, promazine, trimeprazine, and uracil were obtained from Sigma-Aldrich (St. Louis, MO, USA). ACN, EtOH, o-phosphoric acid (o-H_3_PO_4_), sodium hydroxide (NaOH), ammonia (NH_3_), ammonium chloride (NH_4_Cl), and potassium hydrogen phthalate (KHP) were provided by Merck (Darmstadt, Germany).

### 2.2 Apparatus

A Shimadzu (Kyoto, Japan) brand high-performance liquid chromatography (HPLC) instrument was used for all chromatographic analyses. The instrument consisted of a UV detector (SPD-20A), a column oven (CTO-20A), an isocratic pump (LC-20AD), and a degasser unit (DGU-20A3). A Mettler Toledo (Zurich, Switzerland) pH meter and a combined Ag/AgCl glass electrode (Mettler Toledo Inlab 413) were used for pH measurements of the prepared mobile phases. Analyses were performed with ultrapure water provided by a Millipore Direct-Q3 UV water purification system (Bedford, MA, USA).

### 2.3 Chromatographic Analysis Conditions

The analysis of the compounds on the HPLC column was determined in binary mixtures of ACN–water and EtOH–water containing 50, 55, and 60% (v/v) organic solvents at varying pH values (5.5–11.5). Buffer compounds containing 30 mM o-H_3_PO_4_ and NH_4_Cl at the same concentration were used in the mobile phases according to the pH values at which the buffers were effective.

The pH value of the mobile phase was adjusted by adding a 1 M NaOH solution to the mobile phase containing o-H_3_PO_4_ and by adding concentrated NH_3_ solution to the mobile phase containing ammonium chloride. The pH values of the binary mixtures in this mobile phase were evaluated according to the International Union of Pure and Applied Chemistry (IUPAC) guideline, taking into account the reference pH values obtained from the National Institute of Standards and Technology (NIST).^31–33^ KHP (0.05 mol/kg) was used as a reference standard for electrode calibration in hydroorganic mixtures.^34^ The pH value (pH_x_) of this hydroorganic mixture can be determined by eq 1.

$${\mathrm{pH}}_{x}={\mathrm{pH}}_{s}+\frac{E_{s}-E_{x}}{(\ln\;10)RT/F}$$ 1

Here, pH_s_ represents the pH of standard buffer solutions, *E*_s_ represents the electromotive force (emf), while pH_x_ and *E*_x_ represent the pH and emf values of the carrier electrolyte, respectively. According to IUPAC guidelines, pH measurements are standardized using potentiometric systems that rely on a primary standard reference.^31–33^

Solutions of the investigated compounds in binary mixtures were prepared daily. Standard solutions at 100 μg/mL concentrations were prepared in the studied mobile phase. Fresh solutions were prepared daily and stored at +4 °C in the dark until analysis. For testing, a 20 μL sample was injected three times into the HPLC instrument. The average *t*_R_ was calculated from these replicates, showing precision with a relative standard deviation (%RSD) of approximately 0.01.

Before RPLC analysis, the spectral behavior of each compound was determined using a UV–vis spectrophotometer over the range of 190–400 nm to identify the wavelength at which the compound showed maximum absorbance. Based on this analysis, a wavelength of 254 nm was chosen as the maximum absorbance wavelength for promethazine, promazine, and trimeprazine, and the detector of the liquid chromatograph was set to this wavelength.

This liquid chromatographic study was performed at 25 and 37 °C. Chromatographic determination of the compounds was performed on a Kinetex EVO C18 (Phenomenex, 150 × 4.6 mm ID, 5 μm) core–shell column at a constant flow rate of 1 mL/min. There are no data in the literature regarding the determination of the retention behavior of compounds by analysis with HPLC columns produced with the core–shell technique. Additionally, the theoretical plate number of the column being 2000 and above and the tailing factor being below 2 indicate optimum column performance and satisfactory peak symmetry.

### 2.4 Capacity Factor Determination

In HPLC, the capacity factor (*k*) is calculated by considering the dead time (*t*_0_). The *t*_0_ value used for calculating the capacity factors of promethazine, promazine, and trimeprazine was determined by using a uracil solution (0.01%, w/v). In this study, the retention times of uracil and the compounds were determined by averaging measurements from three injections. These average retention times were then used to calculate the k value.

### 2.5 Data Treatment

The *t*_R_ values of promethazine, promazine, and trimeprazine for the study on the HPLC column were determined by using the RPLC method at different pH values in binary mixtures. The _s_^s^p*K*_a_ values of the ionizable and molecular forms (BH^+^, B) were calculated using the nonlinear regression program (NLREG).^35^

### 2.6 Determination of _w_^w^p*K*_a_ Values

The _w_^w^p*K*_a_ values of promethazine, promazine, and trimeprazine in a water medium were determined by evaluating the obtained _s_^s^p*K*_a_ values of the compounds with the *X*_solvent_ – _s_^s^p*K*_a_ ^3,34^ and the reciprocal of the solvent’s dielectric constant (1/ε) – _s_^s^p*K*_a_ relationships.^8^

### 2.7 Green Analytical Chemistry Metrics

The greenness of the solvents and the overall environmental impact of the developed methods were evaluated using GSST, AGREE, and GAPI. Furthermore, unlike conventional green metrics that focus on environmental impact, the BAGI was used to demonstrate the applicability of the methods.^26–30^

## 3 Results and Discussion

Phenothiazine rings have different pharmacological activities depending on their positions substituted in rings 2 and 10, and they are widely used in biological, chemical, and medical research. However, existing literature lacks sufficient data on the p*K*_a_ values, especially for phenothiazines with an alkyl piperazine group. Additionally, limited information is available about how the temperature influences the retention behavior of these compounds. Temperature is an important operating parameter in the RPLC method because it affects both separation efficiency and selectivity in the method.^6,14^ Because p*K*_a_ values in RPLC are influenced by temperature, the pH is also temperature-dependent and must be expressed accordingly. This situation also affects the *t*_R_ value of the analyte. In short, if the p*K*_a_ value of the ionized compound is temperature-dependent, then a change in temperature, even at constant pH, can affect the chromatographic retention value of the analyte.

In the optimized RPLC approach, the mobile phase pH range was selected so that the compounds under investigation would be fully ionized or neutral, considering the p*K*_a_ ± 2 range. This way, the *t*_R_ values of the compounds could be more accurately evaluated across different pH levels. The working pH range of the selected HPLC column in this study is 1–12. Because the tertiary amine in the structure of the three compounds has a p*K*_a_ value of about 9.0 in water, the initial pH of the study could have been 7.0. However, adding an organic solvent to the mobile phase decreases its polarity and enhances its elution strength. Therefore, since the p*K*_a_ values of the compounds could be below 9.0, pH 5.5 was selected as the initial pH.

In this study, the retention time values of promethazine, promazine, and trimeprazine were determined by analysis with mobile phases prepared as binary mixtures containing increasing EtOH and ACN concentrations at physiologically relevant temperatures (37 °C) and room temperature (25 °C) with three replicate analyses. An increase in *t*_R_ values was observed as the mobile phase pH values increased. Sigmoidal graphs showing the changes in the *t*_R_ values of compounds against mobile phase pH (pH_x_), depending on solvent and temperature changes, were prepared. In addition, attention was paid to standardize the pH by considering the surrounding temperature in the binary mixtures examined.^31–33^ Sigmoidal graphs prepared according to the analysis results performed at body temperature (physiologically relevant temperature, 37 °C) are given in Figure 2, and graphs prepared at 25 °C are presented in Figure S1.

**Figure 2:**
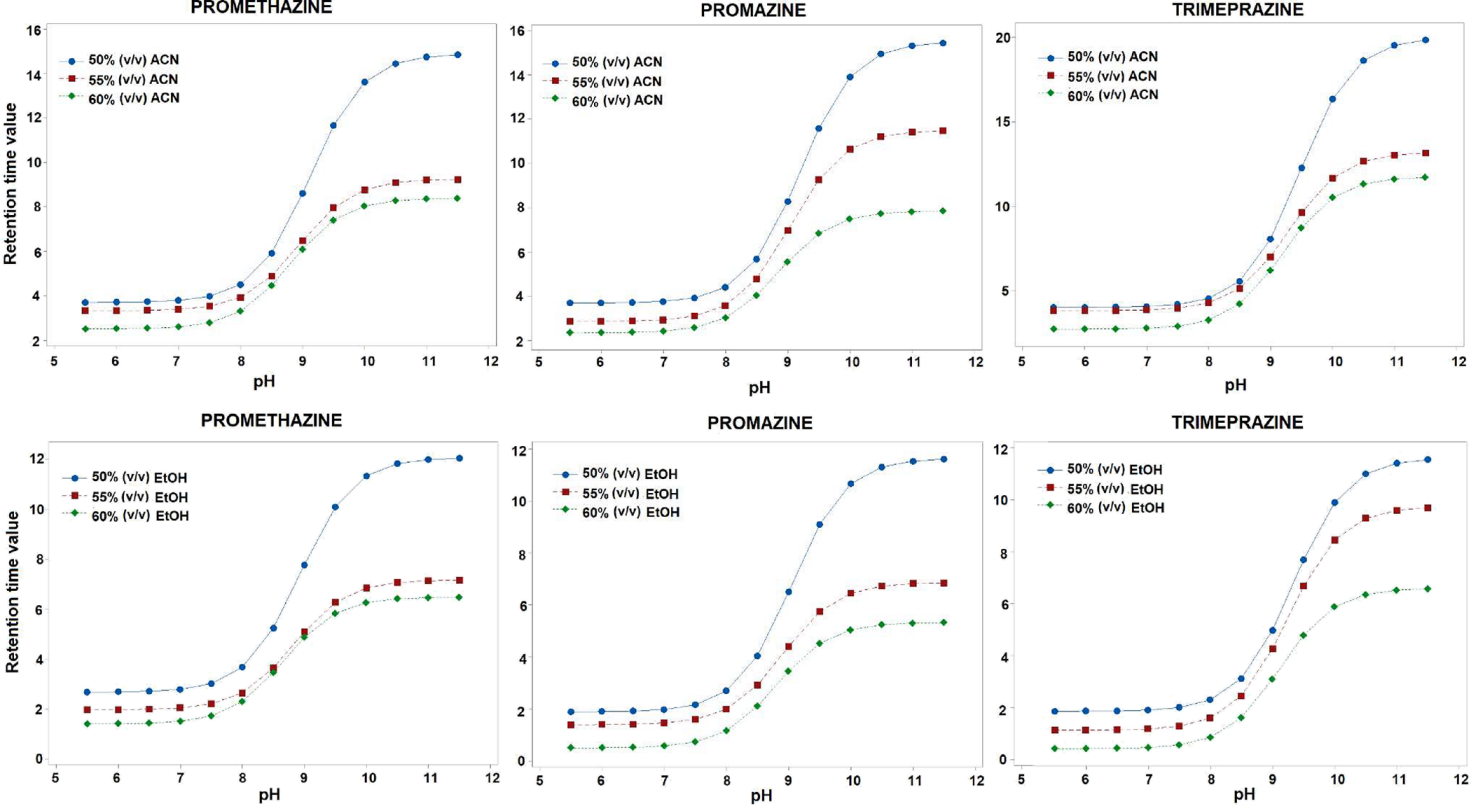
Sigmoidal relationship between *t*_R_ values and pH of the mobile phase in the hydroorganic mixture at 37 °C.

As can be seen from Figure 2, no significant increase in *t*_R_ values was observed due to the temperature increase, especially between pH 5.5 and 8.0. Significant changes were observed in the *t*_R_ values of analytes between pH 8.5 and 11.5. Compounds interacted more with the column in the molecular form (B) beyond 2 units of their p*K*_a_ values. This agreement is consistent with the literature data for basic compounds.^36–39^ Although the chromatographic behavior in liquid chromatography was similar for the compounds in both methods, the green RPLC method produced lower *t*_R_ values. In ACN–water binary mixtures at temperatures of 25 and 37 °C, the viscosity decreased as the amount of ACN in the mobile phase increased ranging between 0.10 and 0.15.^40^ Similarly, in EtOH–water binary mixtures at the same temperatures, the viscosity decreased with increasing EtOH content, ranging from 0.50 to 0.80.^41^ According to literature data, the viscosity in EtOH–water mixtures decreases more significantly with increasing temperature than in ACN–water mixtures. The higher viscosity in the EtOH/water mixture also leads to increased pump pressure in the chromatographic system. In addition, the solvent strength of EtOH (3.6) is higher than that of ACN (3.2). Consequently, analyses with EtOH can be performed at lower volumes than those with ACN. In this study, due to the very low *t*_R_ values, particularly between pH 5.5 and 8.0, and the k values being close to zero, it was not possible to conduct studies at EtOH volumes lower than the ACN amount in the mobile phase.

Chromatograms showing the ionized and molecular forms of analytes at pH 5.5 and 11.0 at 37 °C in binary mixtures containing 50% (v/v) organic solvent are shown in Figure 3, and chromatograms depicting the retention behavior at 25 °C are presented in Figure S2. At high pH, basic analytes are in the molecular form, and their ionization is suppressed. This results in increased hydrophobicity and longer retention time in the column. Significant changes in *t*_R_ occur at pH values within ±2 units of the p*K*_a_.^15,42^ In addition, a decrease in the *t*_R_ values was observed as the temperature increased. In this study, an HPLC column is widely used for the qualitative determination of promethazine, promazine, and trimeprazine, especially for basic compounds. This column significantly improves the peak symmetry of analytes at high pH compared with conventional C18 columns. Analysis of these compounds was initially performed on the X Terra C18 (Waters, 150 × 4.6 mm ID, 5 μm) column, which is commonly preferred for the analysis of basic compounds. Chromatograms obtained from hydroorganic mixtures containing 50% (v/v) organic solvent at a pH of 11.0 are shown in Figure S3. Compounds prepared at the same concentration had lower peak areas. Analysis of these compounds showed longer retention times and weaker peak symmetry compared to analyses performed on the selected HPLC column.

**Figure 3:**
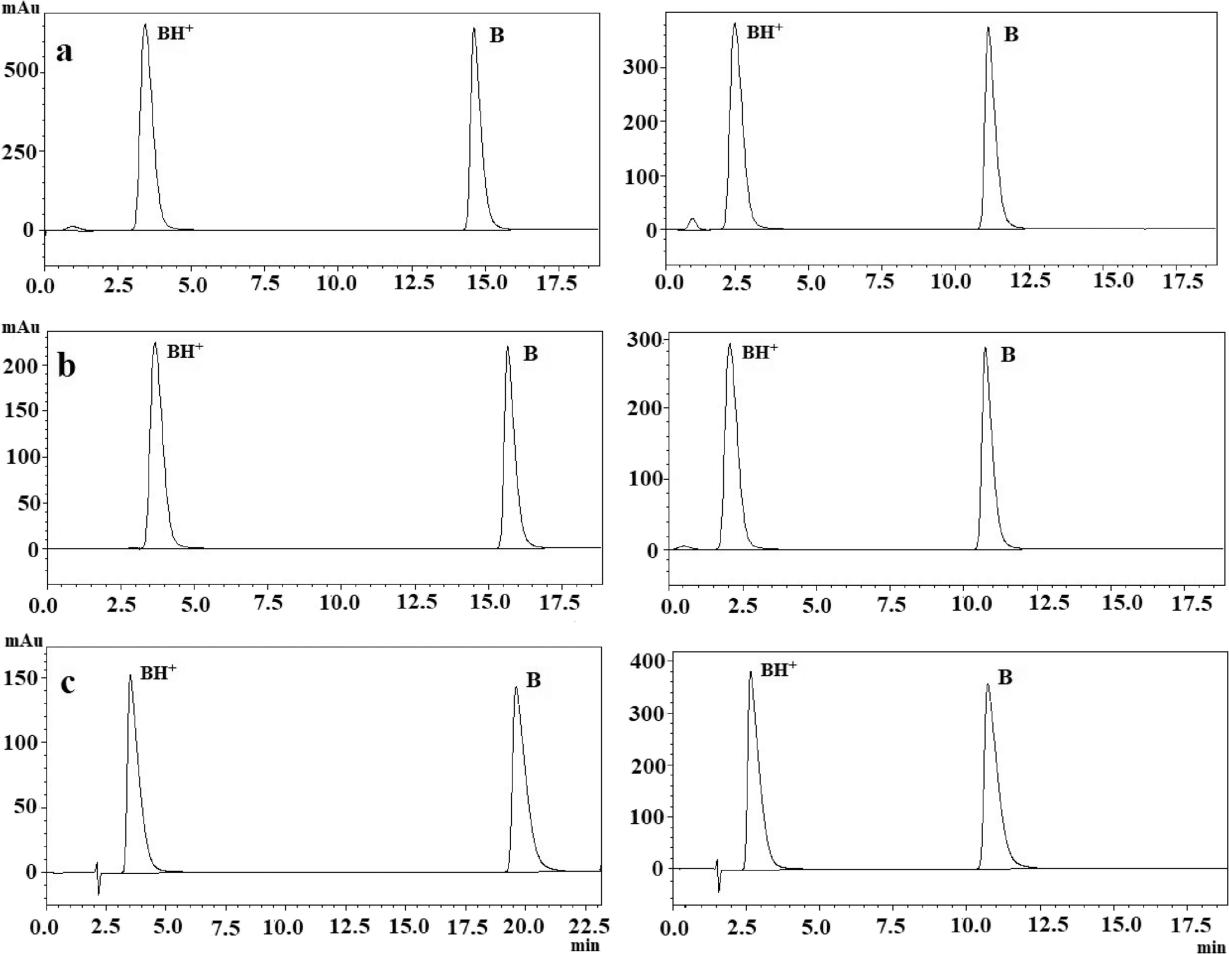
Overlaid chromatograms of compounds (a, promethazine; b, promazine; c, trimeprazine) in binary mixtures containing 50% (v/v) organic modifiers at 37 °C. Left: ACN–water; right: EtOH–water.

The intrinsic *t*_R_ (*t*_RBH^+^_, *t*_RB_) and _s_^s^p*K*_a_ values of the studied compounds were calculated in the NLREG program using the average *t*_R_ values determined depending on the changing EtOH and ACN concentrations and pH values in the mobile phase. The data are shown in Table 1. It is seen that the standard deviation values of the sigmoidal fitting parameters (*t*_RBH^+^_, *t*_RB_, and _s_^s^p*K*_a_) given in Table 1 are very small. Since good results were obtained by using the *t*_R_ value directly, the k value was not used. Although the changes in _s_^s^p*K*_a_ values according to the solvent composition in the mobile phase used were different for each compound, the same type of sigmoidal behavior was obtained as in Figure 3. The _s_^s^p*K*_a_ of basic compounds decreases mainly due to electrostatic interactions that contribute to the p*K*_a_ value. Since ACN (0.46) and EtOH (0.65) have lower relative polarity compared to water (1.00),^42^ the solute–solvent polar interaction decreases as the amount of organic solvent in the mobile phase increases. Therefore, the *t*_R_ value increases as the pH value of the mobile phase increases, while the *t*_R_ value decreases as the amount of organic solvents in the mobile phase increases. In the mobile phase rich in ACN (0.25 ≤ *X*_ACN_ ≤ 0.33) and EtOH (0.26 ≤ *X*_EtOH_ ≤ 0.35), preferential solvation with water exists, and this can explain the decrease in the _s_^s^p*K*_a_ values of the studied compounds when the amount of organic solvent in the mobile phase increases.^43,44^

**Table 1:** Retention Times (*t*_RBH^*+*^_, *t*_RB_) and _s_^s^p*K*_a_ Values Determined at Two Different Temperatures

		promethazine			promazine			trimeprazine		
organic modifier percentage (v/v)	compounds temperature (°C)	_s_^s^p*K*_a_	*t*_RBH^*+*^_	*t*_RB_	_s_^s^p*K*_a_	*t*_RBH^*+*^_	*t*_RB_	_s_^s^p*K*_a_	*t*_RBH^*+*^_	*t*_RB_
50% ACN	25	9.167 (0.064)^a^	3.783 (0.022)	16.204 (0.005)	9.265 (0.088)	4.111 (0.062)	17.935 (0.023)	9.643 (0.078)	4.421 (0.050)	21.961 (0.065)
	37	9.108 (0.045)	3.701 (0.045)	14.887 (0.094)	9.195 (0.036)	3.704 (0.011)	15.509 (0.009)	9.470 (0.073)	4.041 (0.045)	19.999 (0.080)
55% ACN	25	9.007 (0.064)	3.726 (0.070)	10.284 (0.016)	9.096 (0.012)	3.869 (0.051)	13.010 (0.017)	9.527 (0.038)	4.114 (0.009)	15.033 (0.074)
	37	8.947 (0.097)	3.323 (0.070)	9.256 (0.010)	9.042 (0.082)	2.873 (0.071)	11.510 (0.036)	9.329 (0.050)	4.012 (0.031)	13.451 (0.093)
60% ACN	25	8.868 (0.075)	3.160 (0.041)	9.363 (0.068)	8.867 (0.009)	3.084 (0.064)	9.913 (0.014)	9.425 (0.062)	3.958 (0.067)	14.007 (0.061)
	37	8.806 (0.053)	2.502 (0.095)	8.395 (0.009)	8.857 (0.082)	2.361 (0.047)	7.871 (0.033)	9.202 (0.047)	2.746 (0.019)	11.770 (0.066)

		_s_^s^p*K*_a_	*t*_RBH^*+*^_	*t*_RB_	_s_^s^p*K*_a_	*t*_RBH^*+*^_	*t*_RB_	_s_^s^p*K*_a_	*t*_RBH^*+*^_	*t*_RB_
50% EtOH	25	9.117 (0.011)^a^	2.811 (0.054)	13.416 (0.077)	9.185 (0.042)	2.742 (0.016)	14.382 (0.028)	9.388 (0.012)	3.266 (0.073)	13.913 (0.053)
	37	8.926 (0.082)	2.681 (0.094)	12.078 (0.037)	9.048 (0.024)	1.902 (0.071)	11.647 (0.016)	9.329 (0.034)	2.990 (0.033)	11.417 (0.067)
55% EtOH	25	8.999 (0.008)	2.357 (0.042)	8.607 (0.037)	8.978 (0.008)	1.735 (0.070)	7.641 (0.027)	9.278 (0.016)	2.789 (0.088)	11.127 (0.074)
	37	8.824 (0.009)	1.972 (0.034)	7.182 (0.066)	8.914 (0.053)	1.406 (0.067)	6.874 (0.088)	9.243 (0.091)	2.357 (0.037)	9.800 (0.022)
60% EtOH	25	8.900 (0.071)	1.245 (0.009)	5.387 (0.033)	8.808 (0.042)	0.813 (0.072)	6.546 (0.061)	9.167 (0.034)	2.032 (0.055)	7.781 (0.027)
	37	8.666 (0.031)	1.401 (0.062)	6.493 (0.018)	8.805 (0.066)	0.509 (0.075)	5.344 (0.091)	9.118 (0.045)	1.739 (0.064)	7.088 (0.018)

a Standard deviation value.

The absorption, distribution, and excretion (ADME) of ionizable drug active ingredients are greatly affected by p*K*_a_ values.^36^ For this, it is necessary to know the p*K*_a_ (_w_^w^p*K*_a_) values of these drugs in an aqueous environment. To determine these values, studies are carried out on experimental p*K*_a_ values, as well as computational programs. There are a few experimental studies determining _w_^w^p*K*_a_ values for the analytes selected in this study. The solubility (logS) values of promethazine, promazine, and trimeprazine in the aqueous medium are −5.21, −4.94, and −5.11, respectively.^45^ Due to the moderate solubility of the compounds, the chromatographic behavior and _s_^s^p*K*_a_ values in hydroorganic mixtures were determined using different solvents. Because the p*K*_a_ determination cannot be performed directly in water, compound analyses are mostly carried out in a mixture of water and organic solvents. Ternary solvent mixtures are preferred for analyses that require more complex solvent systems than binary mixtures. Because pH control in ternary solvent mixtures is challenging, binary mixtures are generally preferred for isocratic studies. The polar protic EtOH and polar aprotic ACN used in this study dissolve compounds with limited solubility when mixed with water, enabling their chromatographic analysis.

In this study, the _w_^w^p*K*_a_ values of the examined compounds were determined based on the mole fraction of solvent-_s_^s^p*K*_a_ (*X*_solvent_ – _s_^s^p*K*_a_) and 1/ε_solvent_ – _s_^s^p*K*_a_ approaches. In the calculation using the solvent mole fraction, the intercept values of the equation obtained from the linear relationship between the organic solvent *X*_solvent_ and _s_^s^p*K*_a_ give the _w_^w^p*K*_a_ values of the compounds examined. Since the *X*_solvent_ value does not depend on temperature, these values for 50, 55, and 60% (v/v) EtOH were taken from Altun,^46^ and the values for the same percentage of ACN were taken from Barbosa et al.^47^

Equations showing this linear relationship are shown in Table S1. To confirm the computed _w_^w^p*K*_a_ values, an alternative approach was employed. Within the scope of the study, the correlation between the inverse of the dielectric constant value of the organic solvent (1/ε) and the _s_^s^p*K*_a_ values of the compounds was utilized. Since the ε values depend on the temperature, these values were taken from Gagliardi et al. and Navarkhele and Navarkhele for the two temperatures under study.^48,49^ In the obtained linear equations, ε values in the water environment were used for increasing temperatures, and _w_^w^p*K*_a_ values for the compounds could be determined. The formulas derived using this method are listed in Table 2. There was a decrease in the dielectric constant with the decrease in solvent polarity. This leads to changes in the p*K*_a_ values. _w_^w^p*K*_a_ values calculated by using the obtained equations are shown in Table 3.

**Table 2:** Equations Showing the 1/ε – _s_^s^p*K*_a_ Relationship at Two Different Temperatures

dielectric constant		compounds	temperature 25 °C	dielectric constant		temperature 37 °C	*r*
ε_ACN_	59.125–54.264	promethazine	_s_^s^p*K*_a_ = −184.481 1/ε + 12.467	ε_ACN_	56.678–52.048	_s_^s^p*K*_a_ = −192.023 1/ε + 12.490	0.999
		promazine	_s_^s^p*K*_a_ = −263.120 1/ε + 13.722			_s_^s^p*K*_a_ = −215.470 1/ε + 12.999	0.999
		trimeprazine	_s_^s^p*K*_a_ = −143.630 1/ε + 12.068			_s_^s^p*K*_a_ = −170.443 1/ε + 12.472	0.999
ε_EtOH_	52.26–46.92	promethazine	_s_^s^p*K*_a_ = −98.889 1/ε + 11.001	ε_EtOH_	50.43–45.14	_s_^s^p*K*_a_ = −112.290 1/ε + 11.159	0.999
		promazine	_s_^s^p*K*_a_ = −171.730 1/ε + 12.456			_s_^s^p*K*_a_ = −104.030 1/ε + 11.103	0.999
		trimeprazine	_s_^s^p*K*_a_ = −101.060 1/ε + 11.317			_s_^s^p*K*_a_ = −91.054 1/ε + 11.138	0.999

**Table 3:** pwwKa Data Calculated with Two Different Approaches

compounds	temperature °C	*X*_ACN_ – _s_^s^p*K*_a_	1/ε_ACN_ – _s_^s^p*K*_a_	*X*_EtOH_ – _s_^s^p*K*_a_	1/ε_EtOH_ – _s_^s^p*K*_a_	literature values
promethazine	25	10.056	9.980	9.821	9.761	10.00 ^20^; 9.07 ± 0.08 ^21^; 8.86 ± 0.01 ^21^; 9.05 ^24^
	37	10.006	9.924	9.787	9.716	-
promazine	25	10.468	10.361	10.407	10.302	10.30 ^20^; 8.92 ± 0.09 ^21^; 9.00 ± 0.05 ^21^; 9.37 ^22^; 9.56 ^24^
	37	10.213	10.120	9.836	9.766	9.11 ± 0.03 ^23^
trimeprazine	25	10.268	10.134	10.110	10.049	10.12 ^20^
	37	10.191	10.112	10.027	9.968	-

The negative slope of the linear equations in Table S1 and Table 2 suggests that the apparent _s_^s^p*K*_a_ value decreases as the proportion of organic modifier in the mobile phase increases, reflecting the behavior typical of basic compounds. Figure 4 illustrates the linear relationship between the solvent’s macroscopic constant *X*_solvent_ and _s_^s^p*K*_a_ values. In the calculations for the water medium, the ε values were used as 78.30 for the ACN–water mixture and 79.72 for the EtOH–water mixture at 25 °C; the ε values for 37 °C were 74.84 for the ACN–water mixture and 77.81 for the EtOH–water mixture, respectively.^48,49^ Table 3 shows that the data obtained using two different solvents are consistent. However, the sigmoidal behavior of the compounds resulting from differences in solvent polarity and changes in retention times resulted in very small numerical differences in the p*K*_a_ values. This suggests that p*K*_a_ values are affected by conditions such as the solvent system, dielectric properties, temperature, and ionic strength, including the analytical methods used.^5^

**Figure 4:**
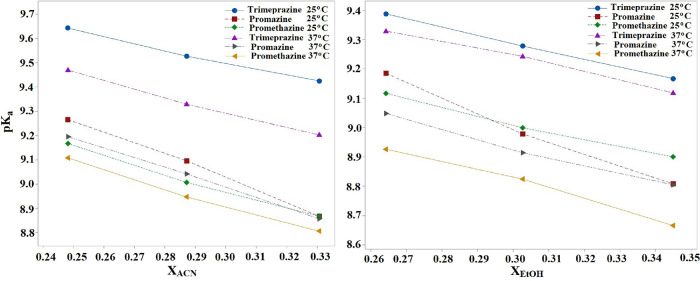
Empirical correlation between p*K*_a_ and the mole fraction of ACN and EtOH.

Drug molecules can ionize at different pH ranges, and the p*K*_a_ value serves as a universal measure of ionization. The ionization process can significantly affect the absorption, distribution, metabolism, excretion, and toxicity of a drug in vivo. Changing the ionic form of a drug can affect its action, absorption, and therapeutic efficacy depending on the pH of the medium.^50^ Therefore, evaluating the degree of ionization of the compounds selected in this study is crucial. Using the Henderson–Hasselbach equation, the percent ionization and p*K*_a_ (at 50% ionization) of the basic functional group of each compound at different pH values were calculated (Figure S4).^51^ Since a green chemistry approach was preferred in this study, the _w_^w^p*K*_a_ values at 37 °C, determined from data obtained from an EtOH-water binary mixture, were used in the calculation of the percentage ionization. Table S2 shows the percentage ionization data calculated for pH values 1–12 using _w_^w^p*K*_a_ values calculated from the EtOH–water binary mixture. At a pH value 2 pH units above the p*K*_a_, the basic compound is in its un-ionized form. It is known that the hydrophilic properties of compounds decrease as the alkyl chain length increases. According to the data in Table S2, the percentage ionization values increase as the alkyl chain length increases.^12^

Currently, the use of solvents such as ACN and MeOH in analytical and sample preparation methods poses significant environmental risks. Based on the principles of green analytical chemistry (GAC), several tools have been developed to assess the “greenness” of newly developed analytical methods. A more precise evaluation of the greenness of analytical procedures can be achieved by applying multiple greenness assessment tools and obtaining generally consistent results across these tools.^27^ In this study, in addition to determining the p*K*_a_ values using the ACN solvent in the conventional RPLC method, a more environmentally friendly method was developed to determine the p*K*_a_ values of the compounds investigated. The greenness of the solvents used in these methods was evaluated using the GSST, and their environmental impacts were assessed using the AGREE and GAPI tools.^26–28^ Additionally, the applicability of analytical methods with white analytical chemistry (WAC) was evaluated with the BAGI tool.^29^

Selecting appropriate green solvents for analytical methods is crucial. For this reason, a novel solvent selection tool developed by Larsen et al. was used.^26^ This tool enables the quantitative evaluation and comparison of solvents based on critical greenness factors. A greenness score (*G*) can be calculated for each solvent. In this study, ACN, EtOH, and water were compared to identify greener alternatives. To determine the greenness scores, the (*G*) values obtained were calculated using the online accessible GSST tool. Accordingly, water, EtOH, and ACN were calculated as 7.3, 6.6, and 5.8, respectively. According to the GSK Solvent Sustainability Guide, *G* ≥ 7 indicates a sustainable solvent, *G* = 5–6 indicates a solvent with limited sustainability concerns, and *G* ≤ 4 indicates a solvent to be avoided. Therefore, water and EtOH, with their high *G* values, are more environmentally friendly compared to ACN.

In this study, as in our previous studies, the environmental impacts of conventional and green RPLC methods were evaluated using the AGREE tool, a new and comprehensive tool for the assessment of greenness.^12^ This tool, which includes 12 principles of GAC, scores the answers to 12 criteria on a scale of 0–1. The results are presented with a pictogram in 12 separate sections in a clockwise direction. The calculated total score is in the middle of this pictogram. The total score is shown as a number between 0 and 1 and a color between red and green.^27^ Accordingly, the conventional RPLC method received a score of 0.55 for ACN, while the green RPLC method received a score of 0.75 (Table 4). The main differences between the two developed methods were determined using the AGREE tool. The first criterion of the AGREE tool evaluates whether the analytical method used can be applied directly without any sample processing. The second criterion asks whether the minimum sample size has been tested. The third criterion asks whether the analytical instrument location is appropriate for sample analysis as straightforwardly as possible. The fourth criterion assesses how many steps are involved in sample preparation. The fifth criterion asks whether the analytical instrument used is automated. The sixth criterion asks whether a derivatization process was performed during sample preparation. The seventh criterion of the AGREE tool addresses analytical waste. The eighth criterion concerns the number of analytes analyzed in a single run and the number of analytes that can be analyzed in 1 h. The ninth criterion concerns the energy consumption of the analytical method under consideration. The tenth criterion concerns the renewable nature of the reagents used. The eleventh criterion addresses the use of toxic solvents in the analytical method. The twelfth criterion discusses the hazards of the reagents for the researcher’s health and environmental safety.^12^

**Table 4:**
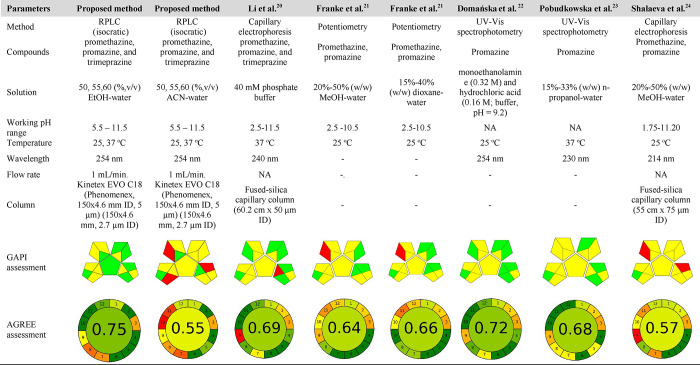
Comparison of the Proposed Analytical Methods with the Selected Methods^a^

a NA, values not available.

The widely used GAPI greenness matrix is employed to evaluate how analytical methods affect the environment. It is a quick, simple, and dependable tool that provides data on the environmental friendliness of the developed method. The pictogram generated with the GAPI tool is colored red, yellow, and green depending on the method used. Qualitative data allow for comparison of methods developed for analytical purposes. The five pentagrams that comprise the GAPI diagram represent various stages of the analytical process, such as sample preparation, sample collection, reagent and solvent use, instrumentation, and the purpose of the analytical procedure.^28^

Unlike conventional green criteria focusing on environmental impact, BAGI considers factors like analysis type, multianalyte detection, techniques, sample processing capacity, prep, throughput, reagent/material choice, preconcentration, automation, and sample size, which are crucial for method applicability. Figure S5 presents BAGI pictograms for both of the developed methods. The color gradient of the pictogram indicates how well the method meets the established criteria: dark blue indicates high agreement, blue indicates moderate agreement, light blue indicates low agreement, and white indicates no agreement. The number within the BAGI pictogram indicates the overall score of the analytical method, ranging from 25 to 100. A score of 25 corresponds to the lowest level of applicability, while a score of 100 indicates excellent performance. Analytical methods scoring above 60 are considered practical. Accordingly, the methods developed in this study are practical in terms of applicability.^29^

There are limited experimental studies aimed at determining the p*K*_a_ values of compounds containing a phenothiazine group. In the study by Sobczak et al., a new phenothiazine derivative was synthesized based on its pharmacological activity.^52^ These compounds were synthesized by substituting different functional groups in the phenothiazine nucleus or by adding carbon or heterocyclic rings to the molecule. In this study, the p*K*_a_ values of fluphenazine and its derivatives were determined by RPLC based on how the *k*_a_ value depends on mobile phase pH. In this study, the compounds were analyzed using a Gemini NX C18 column in the mobile phase using binary mixtures of different percentages of phosphate buffer (pH 2.3–11.5) and ACN (40–55%). The p*K*_a_ values of fluphenazine and its derivatives at ACN concentrations were determined based on the obtained sigmoidal *k* curves as a pH-dependent parameter. These values were then extrapolated to 0% ACN to obtain the aqueous p*K*_a_ values.^52^ This study comprises the only RPLC analyses performed outside of our study. However, the compounds analyzed in this study differ from the chemical structures of promethazine, promazine, and trimeprazine, excluding the main phenothiazine ring structure. Furthermore, there are no studies available on green RPLC analysis that provide accurate pH measurements using pH standardization in binary EtOH–water mixtures. Dugar and colleagues have synthesized several compounds containing a phenothiazine derivative for use in the treatment of Alzheimer’s disease. In silico docking, pharmacokinetic, and toxicity prediction studies met the criteria for a molecule that crosses the BBB. The p*K*_a_ of this compound was estimated using the Jaguar micro- p*K*_a_ tool. The p*K*_a_ of the NH group in the structure was determined to be 11.64.^53^ This synthesized compound differs completely from the side chain attached to the phenothiazine ring in our study.

No study in the literature has directly focused on performing an environmentally friendly analysis to determine the p*K*_a_ values of these three compounds. Therefore, in this study, green/conventional methods developed to determine the p*K*_a_ values of these phenothiazine derivatives were compared with different analytical methods used in the literature to determine the p*K*_a_ values of promethazine, promazine, and trimeprazine using AGREE and GAPI tools (Table 4). There are several p*K*_a_ studies conducted using CE in the literature.^20,24^ The CE method is preferred because it is a popular and more green analytical method. However, the data obtained with the AGREE and GAPI instruments indicate that the developed green/conventional methods are greener than the CE method. Energy consumption (kW) is higher in the CE method than in the RPLC method. Furthermore, the number of analytes in the CE method analyses is higher than that in our study. This data directly reduces the greenness of the methods.

The UV–vis spectrophotometry method presented in the literature analysis and the RPLC methods used for the analysis of three compounds were evaluated with the GAPI tool. The green method developed based on UV spectrophotometry has a higher number of green pentagrams. There are no concerning red pentagrams for these methods. Although the solvents used in Domańska’s study are green, the energy consumption for p*K*_a_ analysis and the large number of analytes are less environmentally friendly than in our study.^22^ In the study by Franke et al., MeOH and dioxane reduced the greenness of the method.^21^ Data were evaluated using GAPI and AGREE pictograms from the literature. Scores above 0.6 on the AGREE assessment tool are considered environmentally friendly and consistent with GAC principles. Table 4 shows the analysis conditions and green pictograms of the methods used to determine the p*K*_a_ values of the compounds studied. These data from the literature were obtained using different analytical methods in various laboratories, and detailed information about the conditions for p*K*_a_ determination is generally not provided. Differences in the solvent, ionic strength, and temperature used in the methods presented in the studies affected the p*K*_a_ values obtained from the methods.

When the analysis results were examined with the two solvents, it was observed that EtOH provided a shorter analysis time for the elution of three compounds from the HPLC column. In the green RPLC method, developed with a GAC approach, 37 °C was selected for the simultaneous determination of the compounds. Once the factors affecting retention behavior, such as the analyte’s p*K*_a_ and lipophilicity constant (log *P*), degree of ionization, ion-interacting agents, chaotropic effects, and secondary interactions, are clearly understood, the elution order can be predicted. Among these, log *P* indicates the hydrophobicity of compounds and determines their elution order from the HPLC column.^15^ The log*P* values for promethazine, promazine, and trimeprazine are 3.14, 3.75, and 4.11, respectively.^45^ In this study, these values are consistent with the elution order of the compounds from the HPLC column.

In liquid chromatography, when analyzing ionized compounds, *k* should fall within the range of 1 and 10, selectivity factor (α) ≥ 1.15, and peak resolution (*R*_s_) ≥ 1.5.^5^ The condition where these parameters were suitable was determined as the optimum separation condition for the EtOH–water mixture containing 55% (v/v) EtOH adjusted to pH 10.5 at 37 °C. The values calculated according to the Purnell equation are given in Table 5. Under these conditions, separation of the three compounds occurred in 9.5 min.

**Table 5:** Calculated Chromatographic Data of Compounds at Optimum Separation Conditions

compounds	*t*_R_	*N*	*k*	α	*k*_2_/1 + *k*_2_	α-1/α	1/4*√N*	*R*_s_
promazine	6.116	5622	2.361					
promethazine	7.074	4011	2.991	1.267	0.749	0.210	15.833	2.497
trimeprazine	9.410	3571	4.526	1.513	0.819	0.339	14.939	4.150
*t*_o_ (uracil)	2.522							

## 4 Conclusions

In this study, the retention behaviors of promethazine, promazine, and trimeprazine in binary mixtures containing phenothiazine groups were determined using green RPLC, a method developed as an alternative to conventional RPLC. The _s_^s^p*K*_a_ values of the compounds at 25 and 37 °C were calculated with the NLREG program using the amount of organic solvent in the mobile phase and the *t*_R_ values with pH changes. Similar sigmoidal behaviors were obtained in three different solvent environments, depending on the substituents attached to the phenothiazine ring of the compounds. Since the data in this study are the first to be determined by both conventional and green RPLC methods, they will contribute significantly to the literature. They will also provide important information on determining the lipophilicity and solubility values of the compounds. According to the GSST tool, EtOH was determined to be greener than ACN. The evaluation conducted using the AGREE and GAPI programs concluded that the method developed using EtOH is more environmentally friendly and more applicable than the ACN solvent developed using the BAGI tool. The separation process was optimized using chromatographic data obtained from this environmentally friendly method. It was concluded that these three analytes can be determined quickly in routine analyses and that the method is suitable for use in real pharmaceutical formulations, stability tests, or biological analyses.

## Supplementary Material



## Notes

Figure S1, sigmoidal relationship between *t*_R_ values and pH of the mobile phase in the hydroorganic mixture at 25 °C; Figure S2, overlaid chromatograms of compounds (a-promethazine, b-promazine, c-trimeprazine) in binary mixtures containing 50% (v/v) organic modifiers at 25 °C; Figure S3, chromatograms of compounds (a-promethazine, b-promazine, c-trimeprazine) in binary mixtures containing 50% (v/v) organic modifier on an X Terra C18 column at 37 °C; Figure S4, % ionization values of compounds at different pH values; Figure S5, BAGI pictograms a) EtOH, b) ACN; Table S1, equations showing the *X*_ACN_ – _s_^s^p*K*_a_ relationship at two temperatures; and Table S2, ionization percentage of the basic functional group for each compound at different pH values (PDF)
