# Supplementary material for: Determination of Retention Behavior and pK a Values of Some Phenothiazines with Green Chemistry Approach
Source: ACS Omega. 2025 Oct 22;10(43):51050–60. doi: 10.1021/acsomega.5c05625 (PMC12593965; doi:10.1021/acsomega.5c05625)
Supplement: Supplementary file 1 [file ao5c05625_si_001.pdf]

SUPPORTING INFORMATION

Determination of Retention Behavior and pK_a Values of Some Phenothiazines with Green Chemistry Approach

Zehra ÖZTÜRK[1], Ebru ÇUBUK DEMİRALAY[2]*, Hülya YILMAZ[3]

[a]*Department of Chemistry, Faculty of Engineering and Natural Sciences, Suleyman Demirel University, Isparta, 32260, Turkey*

[b]*Department of Basic Pharmaceutical Sciences,* Faculty of Pharmacy, *Suleyman Demirel University, Isparta, 32260, Turkey*

[c]*Nanotechnology Research and Application Center (SUNUM), Sabancı University, Istanbul, 34956, Turkey*

**E-mail: ebrucubuk@sdu.edu.tr*

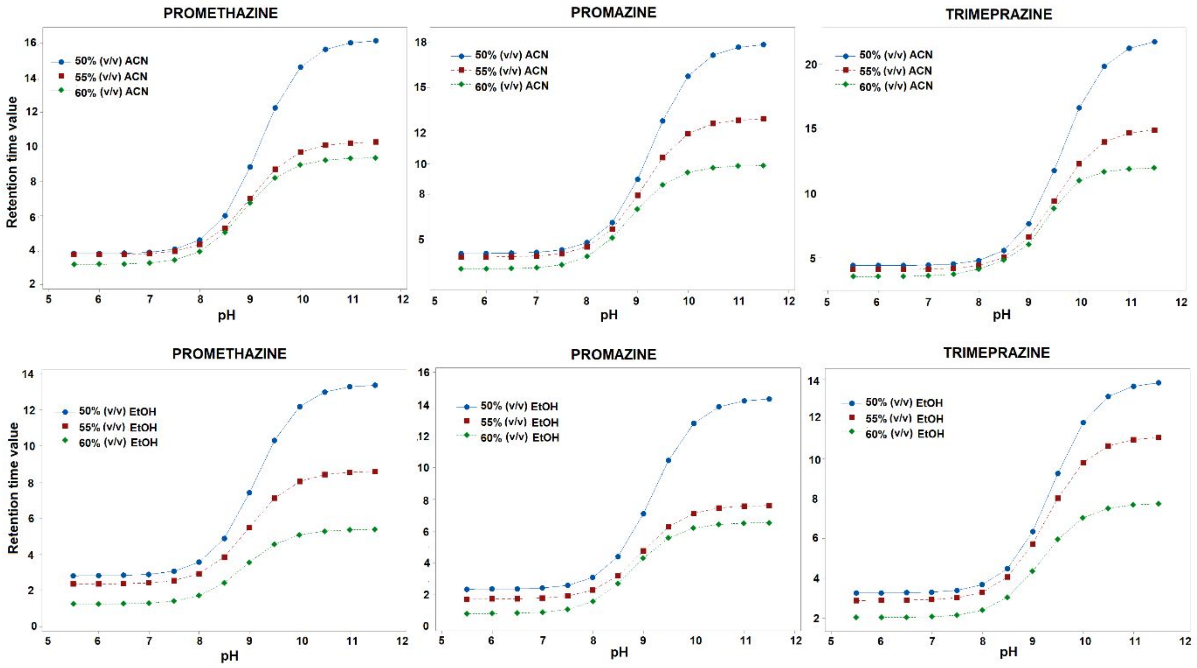

Figure S1. The sigmoidal relationship between t_R values and pH of the mobile phase in the hydroorganic mixture at 25°C.

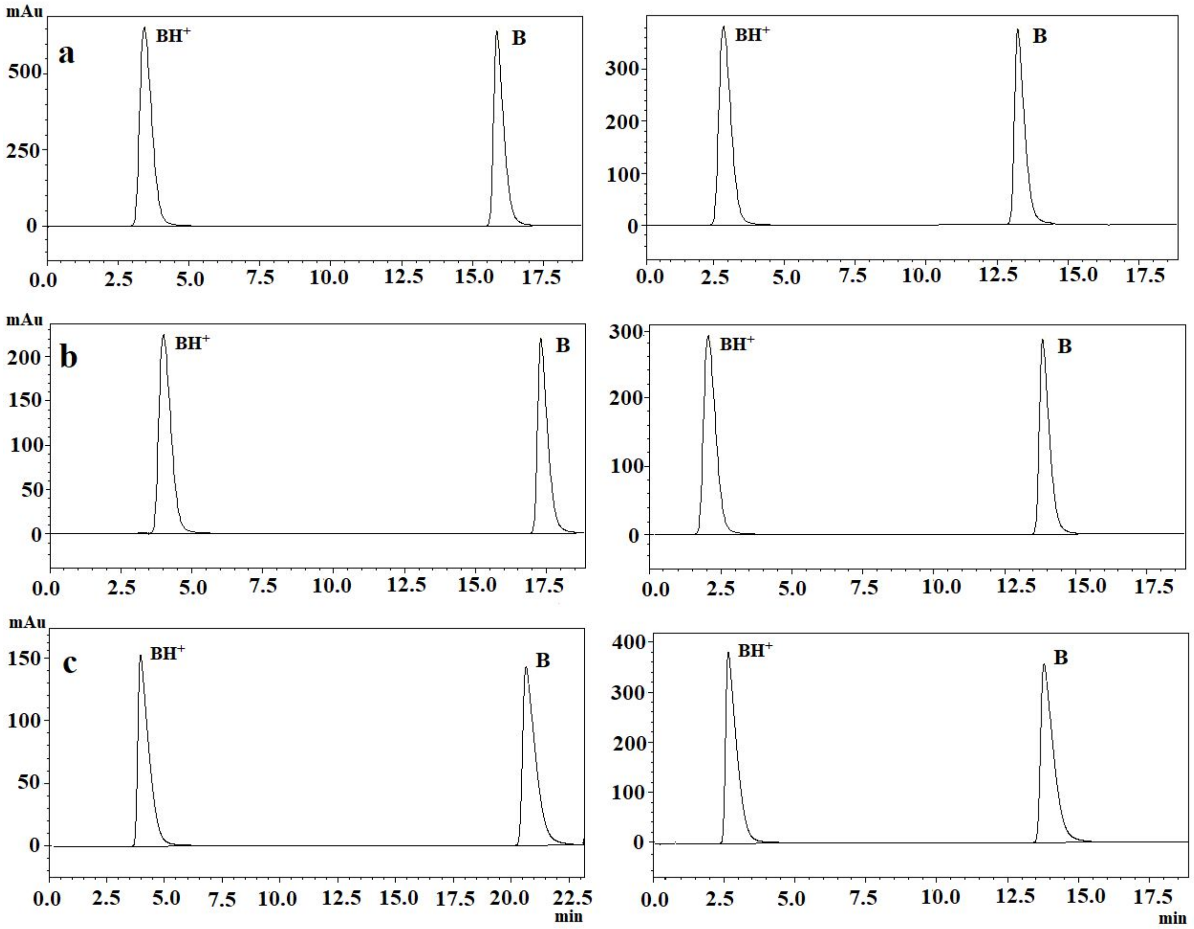

Figure S2. Overlaid chromatograms of compounds (a-promethazine, b-promazine, c-trimeprazine) in binary mixtures containing 50% (v/v) organic modifiers at 25 ^{o}C. left ACN-water, right EtOH-water

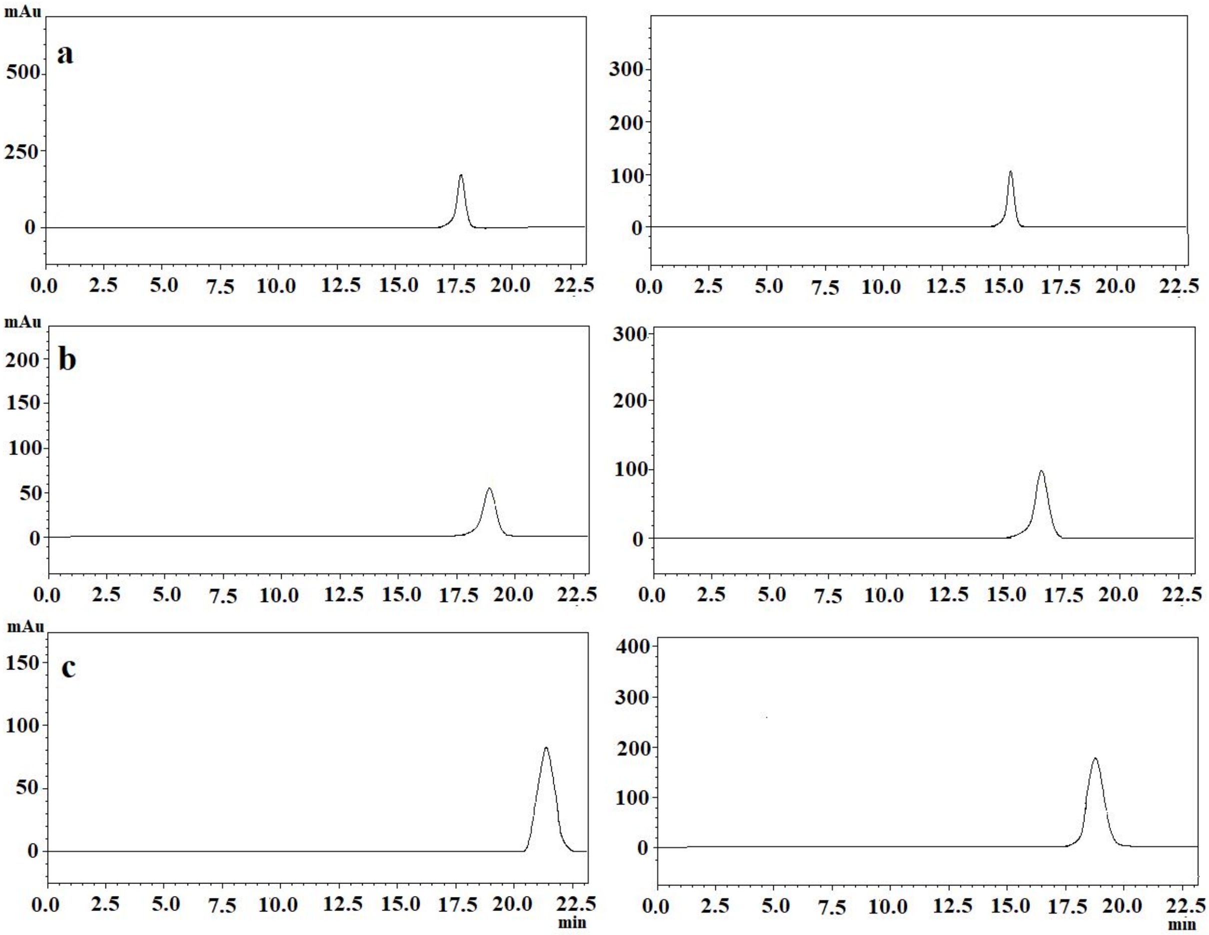

Figure S3. Chromatograms of compounds (a-promethazine, b-promazine, c-trimeprazine) in binary mixtures containing 50% (v/v) organic modifier on an X Terra C18 column at 37 ^{o}C. Left: ACN-water, right: EtOH-water

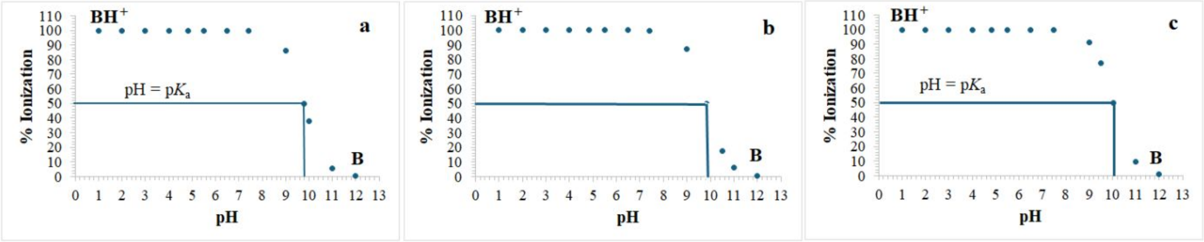

Figure S4. % ionization values of compounds at different pH values a) promethazine b) promazine c) trimeprazine

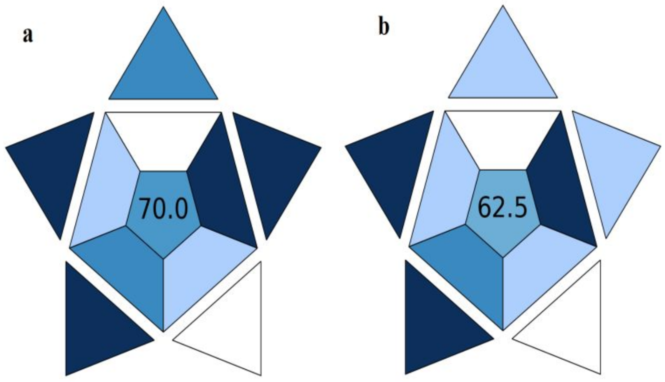

Figure S5. BAGI pictograms a) EtOH, b)ACN

Table S1. Equations showing the X_{ACN} -${}^{s}_{s}pK_a$ relationship at two temperatures

Mole fraction		Compounds	Temperature °C		r
			25	37	
X_{ACN}	0.248-0.331	Promethazine	${}^{s}_{s}pK_a$=-3.609 X_{ACN}+ **10.056**	${}^{s}_{s}pK_a$=-3.645 X_{ACN}+ **10.006**	0.999
		Promazine	${}^{s}_{s}pK_a$= -4.823 X_{ACN}+ **10.468**	${}^{s}_{s}pK_a$= -4.092 X_{ACN}+ **10.213**	0.999
		Trimeprazine	${}^{s}_{s}pK_a$= -2.631 X_{ACN}+ **10.291**	${}^{s}_{s}pK_a$= -3.236 X_{ACN}+ **10.268**	0.999
X_{EtOH}	0.264-0.345	Promethazine	${}^{s}_{s}pK_a$=-2.683 X_{EtOH}+ **9.821**	${}^{s}_{s}pK_a$=-3.231 X_{EtOH} + **9.787**	0.999
		Promazine	${}^{s}_{s}pK_a$=-4.661 X_{EtOH} + **10.407**	${}^{s}_{s}pK_a$=-3.004 X_{EtOH} + **9.836**	0.999
		Trimeprazine	${}^{s}_{s}pK_a$=-2.620 X_{EtOH} + **10.027**	${}^{s}_{s}pK_a$=-2.737 X_{EtOH} + **10.110**	0.999

Table S2. Ionization percentage of the basic functional group for each compound at different pH values

Compounds	% Ionization value				
	pH 2.0	pH 7.4	pH 9.0	pH 10.0	pH 12.0
Promethazine	100	99.591	85.962	37.979	0.609
Promazine	100	99.635	87.269	40.670	0.681
Trimeprazine	100	99.765	91.410	51.554	1.053
